# PLEIOTROPIC HEALTH BENEFITS OF TIME-RESTRICTED FEEDING

**DOI:** 10.1093/geroni/igae098.0583

**Published:** 2024-12-31

**Authors:** Satchidananda Panda

**Affiliations:** Salk Institute for Biological Studies, San Diego, California, United States

## Abstract

The global epidemic of obesity is driving a rapid increase in metabolic syndrome, which in turn contributes to several chronic diseases, including type 2 diabetes, certain cancers, and dementia. To combat this diverse set of diseases, there is an urgent need for simple yet effective measures with widespread benefits across multiple organ systems that can be implemented at a population scale. Time-Restricted Feeding/Eating (TRF or TRE) is a circadian-based dietary intervention where daily energy intake is limited to a consistent 6-12 hour window without explicitly changing the quantity of food consumed. In laboratory animals, this approach sustains circadian expression of the majority of protein-coding genes in a tissue-specific manner. When fed an obesogenic diet, TRF can mitigate metabolic disorders such as adiposity, dyslipidemia, hypercholesterolemia, fatty liver disease, atherosclerosis, and gut dysbiosis. Research from various labs has also shown that this method can reduce the risk and impact of dementia and certain cancers. Genetic and multi-omics studies are beginning to uncover the extensive molecular pathways engaged during time-restricted feeding. This foundational research is likely to drive large clinical trials across diverse populations to assess the feasibility and sustainability of TRE as a population-level intervention to reduce the global burden of diseases.

